# Supersonic gas curtain based ionization beam profile monitor for FLASH proton beam therapy

**DOI:** 10.3389/fonc.2025.1694310

**Published:** 2025-10-27

**Authors:** Milaan Patel, Narender Kumar, Farhana Thesni, William Butcher, Tony Price, Ruth McLauchlan, Carsten P. Welsch

**Affiliations:** ^1^ The Cockcroft Institute, Sci-Tech Daresbury, Warrington, United Kingdom; ^2^ Physics Department, University of Liverpool, Liverpool, United Kingdom; ^3^ School of Physics and Astronomy, University of Birmingham, Birmingham, United Kingdom; ^4^ Department of Radiation Physics & Radiobiology, Imperial College Healthcare NHS Trust, London, United Kingdom

**Keywords:** FLASH therapy, proton beam therapy, diagnostics, non-invasive, ionization profile monitor, supersonic gas jet, beam profile monitor

## Abstract

**Introduction:**

FLASH Proton Beam Therapy (FLASH-PBT) combines the precision targeting ability of proton beam with radiobiological advantage of FLASH effect at ultra-high dose rates (*>* 40 Gy/s) to improve tumor control while reducing the damage to surrounding healthy tissues. The commonly used spot-scanning proton therapy technique relies on real-time beam monitoring to provide feedback to the accelerator for spot switching. This study introduces a novel Supersonic Gas Curtain Ionization Profile Monitor (SGC-IPM) for non-invasive, high-resolution proton beam profile monitoring, aiming to provide real-time feedback to medical accelerators.

**Methods:**

The SGC-IPM uses a supersonic gas jet shaped into a curtain to measure the 2D transverse profile of the beam. Initial tests of the device was conducted on a DC Pelletron accelerator at Dalton Cumbrian Facility (DCF), Whitehaven, UK, followed by later tests on MC40 cyclotron at the University of Birmingham (UoB), UK. Across both the experiments, the device was directly coupled to the vacuum side of the beamlines and beam profiles were recorded for protons at energies ranging from 4–28 MeV and currents ranging from 1–100 nA, with various beam sizes and shapes.

**Results:**

The SGC-IPM successfully measured beam profiles and demonstrated a linear response to beam currents across the measurement range, and its response at different energies was quantified by introducing an energy-dependent detection factor, *D* which is used to quantify the sensitivity of the device. The detector was upgraded after the first set of experiments at DCF resulting in sensitivity improvement by a factor of 80 in later experiments at UoB.

**Discussions:**

A mathematical model is introduced to show that device’s response depends on particle fluence, a quantity independent of dose rate. It’s linear response to beam current is used to extrapolate measurements at conventional dose rates to assess its performance at FLASH dose rates. The performance is evaluated in terms of threshold dose required to measure beam profile for a standard 1-liter clinical volume positioned 15–20 cm deep in water.

**Conclusion:**

This study presents a viable solution for non-invasive proton beam profile monitoring for FLASH-PBT. The device shows a linear response to beam current within the measurement range. The mathematical model quantifies the device’s sensitivity and provides a means to calibrate it for dose estimation.

## 1 Introduction

The basic aim of radiotherapy is to provide the targeted dose to the tumor while minimizing the dose to surrounding organs at risk. One of the promising technique is FLASH Proton Beam Therapy (FLASH-PBT), in which a proton beam is delivered at ultra-high dose rates *>* 40 Gy/s to take advantage of the precise targeting ability of proton beams, along with the FLASH effect (1, 2). Preclinical studies have shown that FLASH radiotherapy improves tumer control probability to normal tissue complication probability ratio (3). Full exploitation of FLASH-PBT requires overcoming challenges in accelerator technology to reliably generate and deliver extremely high beam currents (4), as well as in dosimetry to precisely monitor the dose (5). One of the employed techniques in FLASH-PBT is spot scanning. For example, it is used to deliver the total dose within a single fraction to a small volume (6), and a few pre-treatment QA programs have been reported in the literature based around this approach (7).

Existing proton beam therapy facilities that can deliver FLASH-PBT often use isochronous cyclotrons, synchro-cyclotrons, or synchrotron accelerators (8). Operating these accelerators to deliver FLASH doses requires beam currents on the order of 100–1000 nA, delivered in a few hundred milliseconds to qualify for FLASH dose rates. This also limits the reaction times associated with beam control, thus requiring real-time beam monitoring systems. The control system of a typical spot scanning approach usually rely on ionization chambers for dose monitoring to direct the beam to the next spot after the prescribed dose is delivered to the current spot (9). However, errors introduced by ion recombination effects (10) present significant challenges in implementing these chambers as beam monitors for control systems, as described in a comprehensive study (11). Therefore, fast and accurate beam profile monitoring is crucial to realize FLASH-PBT.

This study presents a non-conventional approach to measure the two-dimensional profile of the beam using a supersonic gas curtain-based ionization profile monitor (SGC-IPM). The device is a modified version of the beam gas curtain (BGC) designed for the LHC (12–14). The SGC-IPM generates a supersonic gas curtain by extracting the core of a free supersonic jet in a vacuum chamber and reshaping it into a gas curtain. The system is attached directly to the accelerator beamline on the vacuum side, with the gas curtain crossing the proton beam in transverse direction. As the ion beam transverses the curtain, it interacts with the gas molecules, ionizing them in the shape of the beam. These ions are then extracted and sampled by a detector to construct the beam profile. The operating principle is similar to that of an ionization chamber, except that ionization occurs locally within the curtain, and extraction is done in the vacuum, minimizing the possibility of interaction with the neutrals. This eliminates the recombination issue associated with gas-filled ionization chambers at FLASH dose rates. The relatively low density of the gas curtain (∼ 10^16^ molecules m^−3^) allows for measurement without perturbing the beam, enabling its use for *in-vivo* conditions. The ion collection times are on the order of microseconds, similar to those of ionization chambers, allowing for fast detection and the resolution can be adjusted to *<* 1*σ* by changing the curtain width.

This study presents the first successful measurement of a proton beam profile with the SGC-IPM, with energies ranging from 4 to 28 MeV and beam currents between 1 and 100 nA. The data presented were recorded for protons from two different accelerator facilities: a DC Pelletron accelerator at the Dalton Cumbria facility, Whitehaven, UK, and the MC-40 cyclotron facility at the University of Birmingham, UK. The paper presents a mathematical model for scaling the detector’s response to different beam currents and energies. This model is used to draw observations of its performance under different beam parameters for both conventional and FLASH clinical beams. The study further discuss current challenges and ongoing efforts to address them in future iterations.

## 2 Methods

This section presents the operating principle of the SGC-IPM, outlines the experimental setup for measuring the beam profile, and provides a list of the beam parameters for which the beam profiles were measured.

### 2.1 Supersonic gas curtain ionization profile monitor

**Figure 1:**
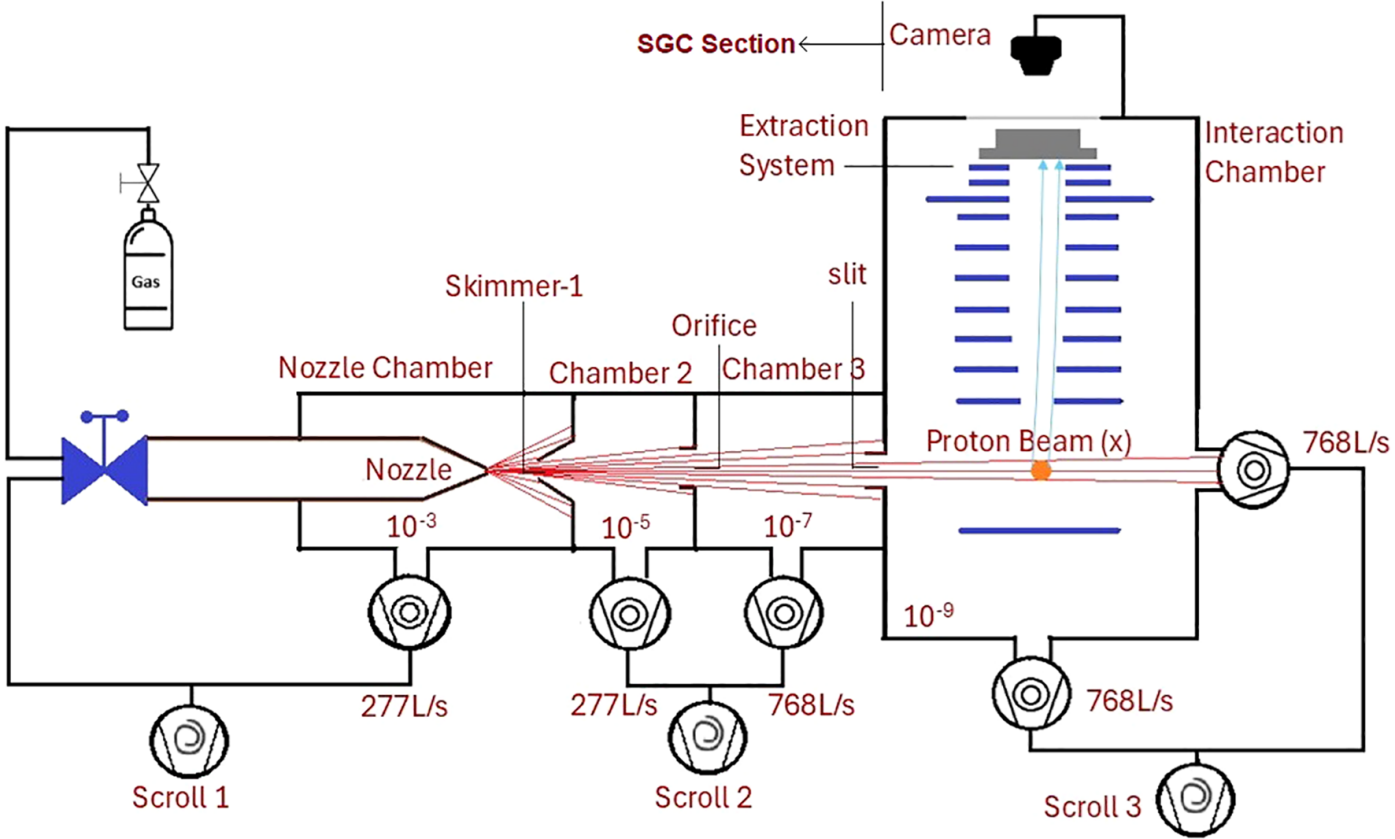
Schematic of the supersonic gas curtain ionization profile monitor (SGC-IPM) looking along the direction of proton beam: Nozzle chamber, chamber 2 and chamber 3 froms the SGC section. The extraction system, detector and camera (installed outside) forms the IPM.

**Figure 1** shows the schematic of SGC-IPM. It can be divided in two parts; the section generating supersonic gas curtain (SGC) and the interaction region housing the ionization profile monitor (IPM).

In the SGC section, high-pressure (5 bar) argon or nitrogen gas is injected into a vacuum of 10^−3^ mbar through a small orifice (30 *µm*) to generate a supersonic gas jet inside the nozzle chamber. At the end of the nozzle chamber is a conical skimmer with a diameter of 0.4 mm, which extracts the supersonic core of the jet into chamber 2. A circular orifice with a diameter of 2 mm is then used to filter the central uniform-density part of the extracted supersonic core to generate a ‘molecular beam’ of gas into Chamber 3. It houses a removable scanning ion gauge (not shown in the figure) to measure the density profile of the molecular beam and tuning its size. The relative positions of the nozzle and skimmers are adjusted to introduce a controlled angular spread of the molecular beam. This brodens the molecular beam just enough to extract a curtain of uniform density through a rectangular slit (20 x 0.4 mm), inclined at 45^°^, mounted at the end of the third chamber. The curtain then enters into the last interaction chamber housing the IPM, maintained at *<* 10^−8^ mbar. The shape and density of the curtain are verified experimentally using scanning ion gauge to ensure that the desired conditions are achieved (15).

In the IPM, the interaction of the proton beam with the gas curtain ionizes the gas atoms. These ions are then extracted from the curtain while maintaining their spatial distribution using an external electrostatic field of ∼10 kVm^−1^ generated by a series of circular electrodes. Proton beams of energy *>* 1 MeV are rigid enough to be affected by this field. The extracted ions are collected on an Microchannel Plate (MCP) detector, which projects the beam profile onto a phosphor screen, and the resulting image is captured using a standard CMOS camera. The shape of the image mimic the beam’s profile and the counts relates to the beam current. The operating principle of the SGC-IPM is described in more detail in our previous work (16). The errors associated with detection process contributing to beam profile measurements has been extensively studied in our previous work (13).

The time required to generate the beam profile can be estimated based on three factors: the detection time for individual ions, the total number of ions needed for statistical significance, and the number of ions detected simultaneously. The detection time for individual ions corresponds to their average transit time from the interaction region to the detector, which is determined by the electrostatic field distribution and is typically of the order of microseconds (17). The total number of ions required depends on the statistical confidence level required to reliably represent the beam shape - for a Gaussian beam with 95% confidence, approximately 1000 ions are necessary (16). The number of simultaneous detections depends on the ionization rate, which in turn is influenced by the beam current and energy, assuming a fixed gas curtain density and thickness. Therefore, for a given configuration of the SGC-IPM, the time taken to generate the beam profile ultimately depends on the beam energy and current. The next section incorporates these dependencies to estimate sensitivity of the device in terms of proton fluence, a quantity independent on the dose rate, which is then used to estimate the performance of the SGC-IPM under FLASH beam conditions.

### 2.2 Experiments

The beam profile measurements were carried out at two different proton beam facilities in the UK, where the SGC-IPM was transported to each facility and coupled directly to the vacuum side of the accelerator. The first set of experiments took place in July 2023 on a DC Pelletron accelerator at the Dalton Cumbria Facility (DCF) in Whitehaven, UK. In this set, measurements were recorded for proton beams of various shapes and sizes with three distinct energies available within accelerator constrains: 4 MeV, 6 MeV, and 8 MeV, with two sets of beam currents: 10 nA and 100 nA. A 99.999% pure tantalum foil mounted downstream of the SGC-IPM was used as a beam dump and a monitor to check stability of the beam current. The absolute value of the current was recorded before and after each measurement using a movable Faraday cup installed upstream of the beamline.

The second set of measurements was carried out at the MC40 cyclotron at the University of Birmingham (UoB) in August 2024. In these experiments, a new detector system was installed in the SGC-IPM to improve sensitivity. This upgrade was intended to reduce the exposure times from a few seconds to a few 100’s of milliseconds. The experiments followed a similar approach to measure proton beam profiles at 10.8, 16, 20 and 28 MeV, and current ranging between 2–71 nA for each set of energy. However, poor beam transport and vacuum conditions resulted in limited beam stability for the most part, allowing useful data to be generated only at 28 MeV.

A Marcus Ionization Chamber installed at the opposite end of the SGC-IPM, was used to set the beam current for the accelerator. However, due to saturation at high beam current, it could only be used below 10 nA. For higher beam currents, a movable Faraday cup installed approximately 2 meters upstream of the SGC-IPM was used instead. To account for beam transport losses between the Faraday cup and the SGC-IPM, a loss factor was calculated by comparing measurements from the Faraday cup and the ionization chamber when the cyclotron was operated at lower current. This loss factor was then applied to Faraday cup measurements at higher currents to estimate the actual beam current reaching the SGC-IPM.

**Table 1** summarizes the range of beam parameters recorded at DCF and UoB. Measurements at DCF were performed using argon and nitrogen gas curtains due to their availability and prior experience in generating uniform curtains. These experiments revealed better performance with argon compared to nitrogen, primarily attributed to the higher density achievable with an argon curtain. Consequently, later experiments at UoB were mostly conducted using argon gas, with nitrogen used only in a few measurements for comparison.

**Table 1:** Beam parameters at which beam profile measurements were recorded using argon and nitrogen curtains on a DC Pelletron accelerator at the Dalton Cumbria Facility (DCF) and the MC40 cyclotron facility at the University of Birmingham (UOB).

Gas Curtain	Energy (MeV)	Current (nA)						
Argon	4	10	98	99				DCF
	6	10	103	102	101			
	8	11	102					
Nitrogen	4	10	101					DCF
	6	10	102	102				
	8	10	99	100	102			
Argon	~11 (10.8)	2.0	4.0	5.9				UoB
	16	5.1	10.3	15.5	20.6	30.9		
	20	6.4	12.8	19.3	25.8	38.7	51.7	
	28	9.1	18.0	26.9	35.8	53.6	71.4	
Nitrogen	28	9.1	18.0	26.9	35.8	44.7		UoB

## 3 Results

This section presents the results of the beam profile measurements. The first subsection discusses the procedure for determining the beam size from the recorded image. The second subsection introduces the mathematical model describing the ‘detection factor’, which relates the detector response to the beam current and energy. The third subsection evaluates the properties of the ‘detection factor’ for different beam current and energies.

### 3.1 Beam profile measurements

To measure the beam profile, the MCP is operated in ion collection mode, with the input channel biased to -2 kV and the output channel grounded. The phosphor screen is set to a positive potential of 3 kV. The extraction system plates are biased to create a linear potential gradient, ranging from zero at the center of the interaction region to the MCP bias potential at the detector. This setup generates a uniform electric field, extracting ions from the interaction region while preserving their relative positions as they are transferred to the MCP detector. The camera at the end of the detector assembly captures an image of the ionization pattern, as seen from the detector axis.

**Figure 2:**
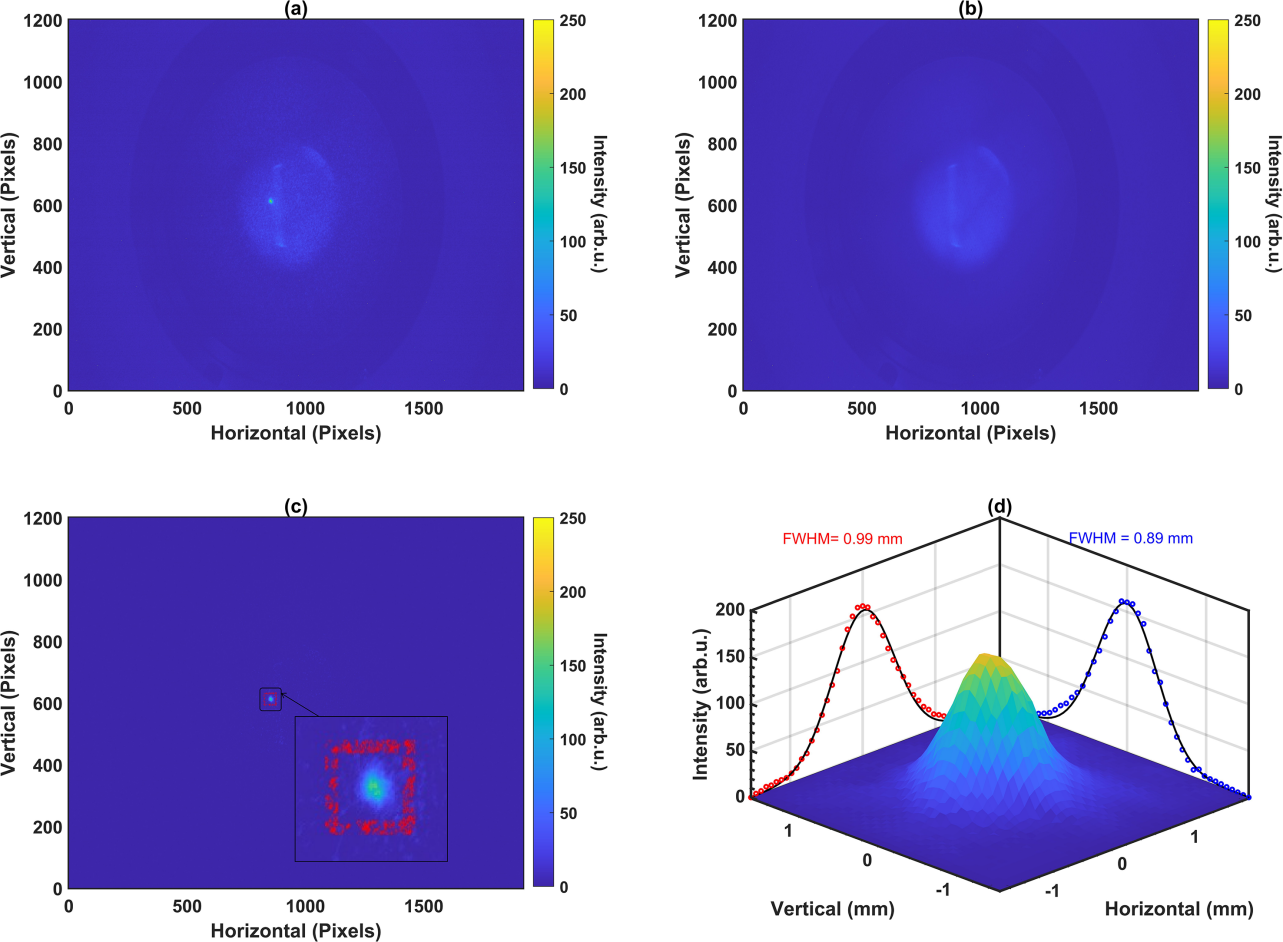
Beam profile measurements for a 6 MeV, 100 nA beam with a 1 s integration time: **(a)** with the gas curtain on, showing the transverse profile along with beam-induced background ionization, **(b)** with the gas curtain off, showing only the beam-induced background ionization, **(c)** after subtracting the images shown in **(b)** from **(a)** with ROI marked in red, and **(d)** beam profile with 2D panels showing the FWHM of Gaussian fits.

A sample image captured by the IPM with both the beam and curtain on is shown in **Figure 2a**. This image corresponds to a 6 MeV proton beam with a current of 100 nA, captured with a camera integration time of 1 second. The circular spot represents the 2D beam profile as projected onto the gas curtain, while the diffused line adjacent to it corresponds to ionization of the background gas along the beam path.

Since the 2D profile originates from ions generated in the gas curtain, their neutral velocity is conserved, introducing a drift along the jet direction during extraction. In contrast, ions from the background gas do not experience this drift, resulting in a slight offset between the 2D profile and the diffused line. This offset helps isolate the 2D profile effectively from the background for beam size smaller than 1 mm, but cannot avoid overlap for broader beams. To address this, a background image is captured with the gas jet switched off, as shown in **Figure 2b**. This background image is subtracted from the original image to remove the contribution of background gas. The subtracted image is shown in **Figure 2c**. In the next step, the region containing the beam profile is cropped and further image analysis is performed to remove saturated pixels. The process is described in our earlier work (18). The 2D beam profile scaled to the absolute length in mm is shown in **Figure 2d**.

**Figure 3:**
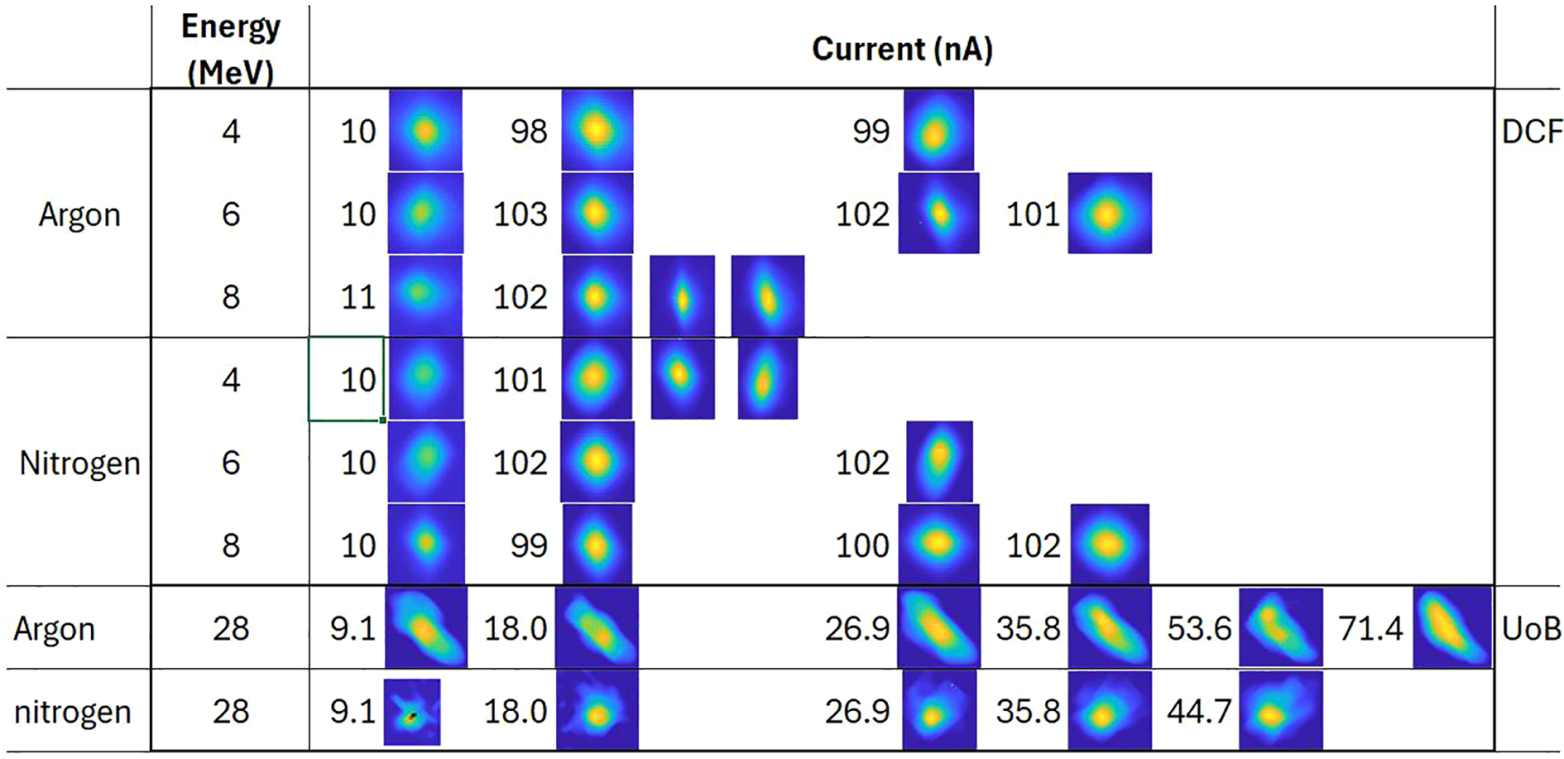
Beam profiles recorded for different beam parameters listed in **Table 1**. Profiles for DCF data are shown with a 1000 ms integration time, while profiles for UoB data are shown with a 500 ms integration time. The color scale is normalized to beam current. The images represent beam shape and are not to scale.

For each beam parameter listed in **Table 1**, images were recorded for at least three different camera integration times, with each set comprising of 100 images. These gives us multiple data sets for every beam parameter from **Table 1**. Some datasets included varying beam shapes and sizes under identical conditions (only for measurements at DCF) to assess the detector’s sensitivity to beam geometry. One of the profiles recorded for various beam parameters are shown in **Figure 3**. The images illustrate the shape of the beam, with the color scale normalized to beam current. At beam energies of 10.8, 16, and 20 MeV the clear beam profiles could not be measured with reasonable accuracy because of low signal-to-noise ratio due to unstable beam discussed earlier along with poor vacuum (*>* 10^−7^ mbar) and hence are not shown here.

### 3.2 Detector response with beam energy and current

The detector response is quantified by the total number of counts registered on the pixels, which depend on the ions generated in the interaction region. Ion production is influenced by the probability of ionization, which varies with both beam current and energy. Thus, an independent parameter is identified to relate the counts to beam current and energy. To introduce this concept, consider a beam with a particle flux that follows a Gaussian distribution, *f* (*x, y*), in the *x*, *y*-plane centered at the mean value, as shown in Equation 1.


(1) $$f(x,y)=Aexp(-(\frac{x^{2}}{2\sigma_{x}^{2}}+\frac{y^{2}}{2\sigma_{y}^{2}}))$$

**Figure 4:**
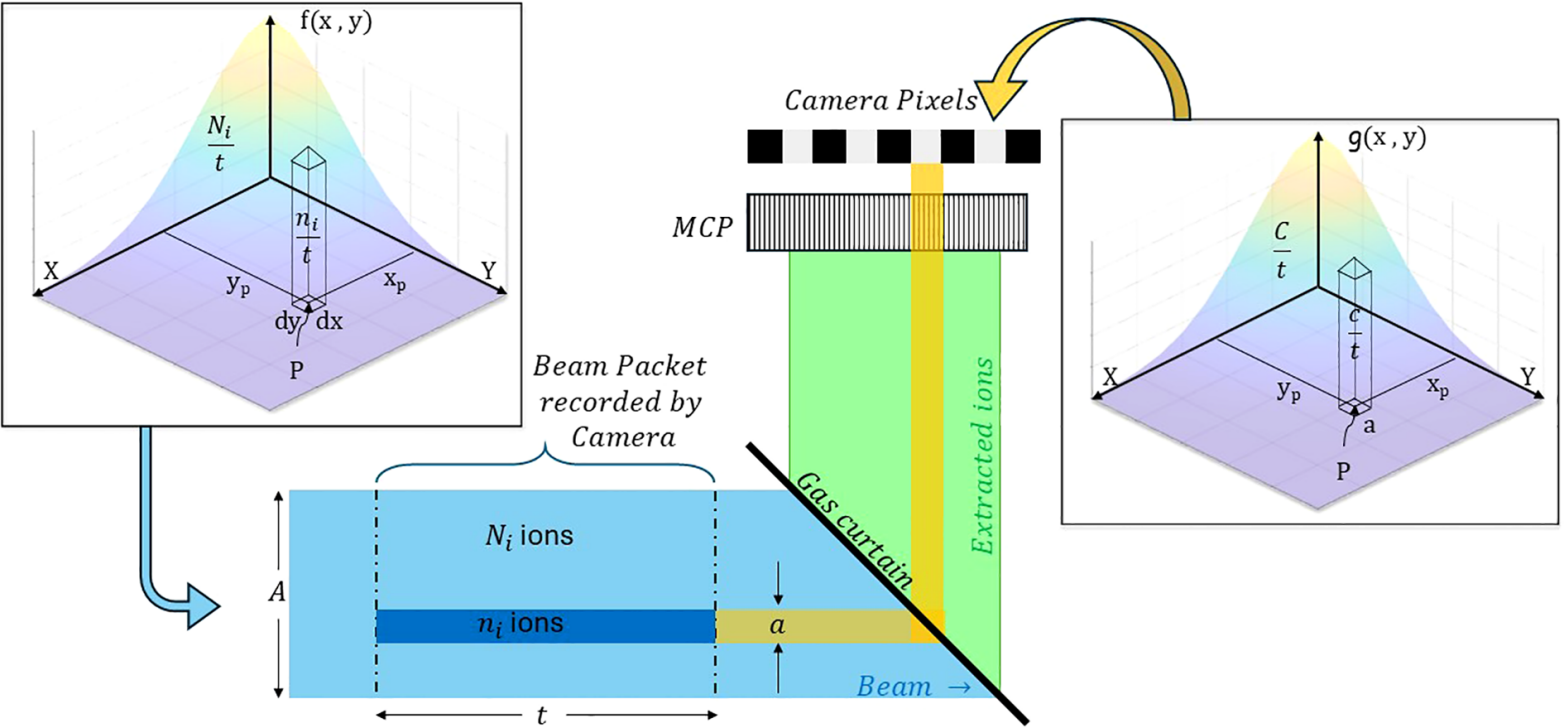
Depiction of beam interaction with the gas curtain, with colour bands representing protons and ion trajectories. Yellow bands represent the pixel area *a* projected onto the curtain and its transformation onto the beam cross-section *A*. The dark blue volume illustrates the beam ions contributing to the flux through *a*, depicted in the left inset as a volume element within the Gaussian flux distribution (quarter section), with the right inset showing the resulting count distribution.

The value of *f* (*x, y*) at any position (*x, y*) represents the ion flux in particles/(mm^2^ × s), and its integral would represent the total number of ions/s. The recorded beam profile on the image plane of the camera follows a distribution function g(x,y) whose values represent the counts/(pixel × s). A relationship between *f* (*x, y*) and g(x,y) can be established based on the detection principle illustrated in **Figure 4**.

When the beam crosses the curtain, it ionizes the curtain atoms, forming a projection of its profile on the curtain plane. If these ions are collected on the detector following straight trajectories, they form an image of the beam on the detector plane identical to the original profile. The beam profile recorded by the camera at the end of the detection system is in discretized form, with each discrete point corresponding to the size of an individual detector pixel. Since the curtain is inclined at 45°, the area projected by a particular pixel on the beam’s cross-section matches the area occupied by that pixel on the recorded beam profile. Assuming a linear progression of particles without random movement, the distribution function of the beam profile measured by the detector, g(x,y) , follows the same functional form as f(x,y) , scaled by a factor β that can be decomposed into dimensionless factors specific to the detector and probability of ionization, as shown in Equation 2.


(2) g(x,y)=β·f(x,y)

$$where,\;\beta =G_{c}\cdot E_{L}\cdot QE\cdot G_{MCP}\cdot E_{OAR}\cdot E_{IPM}\cdot P_{ion}(E)$$

Here, $G_{c}$ is the camera gain, $E_{L}$ is the light coupling efficiency of the camera, QE is the quantum efficiency of the phosphor screen, $G_{MCP}$ is the MCP gain, $E_{OAR}$ is the optical area ratio of the MCP, $E_{IPM}$ is the extraction efficiency of the IPM, and $P_{ion}(E)$ is the probability of ionization.

The counts registered by a single pixel of area a within the measured profile can then be estimated from the total number of ions crossing its projected area (which is also a ) on the beam’s cross-section during the measurement time. The total number of ions, $n_{i}$ , crossing the projected area of the pixel P located at $\left(x_{p},y_{p}\right)$ is then given by the surface integral of f(x,y) over the pixel area, multiplied by the exposure time t of the camera. This is shown as the insets of **Figure 4**. The total number of counts, c , is then expressed as follows:


(3) $$c=\beta \cdot n_{i}=\beta \cdot (\int_{x_{p}-dx/2}^{x_{p}+dx/2}\int_{y_{p}-dy/2}^{y_{p}+dy/2}f(x_{p},\;y_{p})\text{d}x\text{d}y\cdot t)$$

By discretizing the above equation for individual pixels, *dx* × *dy* can be approximated as *a*. The integral can be expressed as the product of the value of the function at location *P*, $f(x_{p},y_{p})$ , and the pixel area *a*:


(4) $$c=\beta \cdot (f(x_{p},y_{p})\cdot a\cdot t)$$

A similar approach can be applied to calculate the total counts, *C*, registered by the camera over the integration time, *t*, by integrating over the entire beam area, *A*. This is expressed as follows:


(5) $$C=\beta \cdot (\iint_{A}f(x,y)\text{ d}x\text{d}y\cdot t)$$

The integral in this equation represents the beam intensity, which can be estimated using the instantaneous beam current, *I*, and the charge, q which simplifies Equation 5 and express the value of β as


(6) $$\beta =\frac{C}{\frac{I}{q\;e}\cdot t}$$

Substituting the value of *β* from Equation 6 into Equation 4 and rearranging it yields the ion flux per unit count on pixel *P* expressed in terms of the beam current, integration time, and the total counts registered by the detector.


(7) $$\frac{f(x_{p},y_{p})\cdot t}{c}=\frac{\frac{I}{q\;e}\cdot t}{C}\cdot \frac{1}{a}$$

Equation 7 represents the threshold value of beam fluence to generate sufficient ionization in the gas curtain to register a single count on any pixel of the camera. Simply put, the left-hand side of Equation 7 quantifies the sensitivity of the detector; therefore, we define it as the ‘Detection Factor’, *D*.


(8) $$D=\frac{\frac{I}{q\;e}\cdot t}{C}\cdot \frac{1}{a}=\frac{1}{\beta \;a}$$

Equation 8 shows the relation between D and the scaling factor β . Since, β is a function of the beam energy, it makes D also a function of the beam energy. Thus detector response can be related to the beam energy using the parameter D , and to the beam current using the total registered counts C . D is expected to follow a similar trend as probability of ionization while C varies linearly with the beam current at a given energy. For a fixed gas jet density and beam current, $P_{ion}(E)\propto \sigma_{i}$ , ionization cross section. Hence, the response of detector for unknown beam energy should ideally scale similarly as the ionization cross-section scales with energy.

### 3.3 Properties of the detection factor and integrated counts

**Figure 5:**
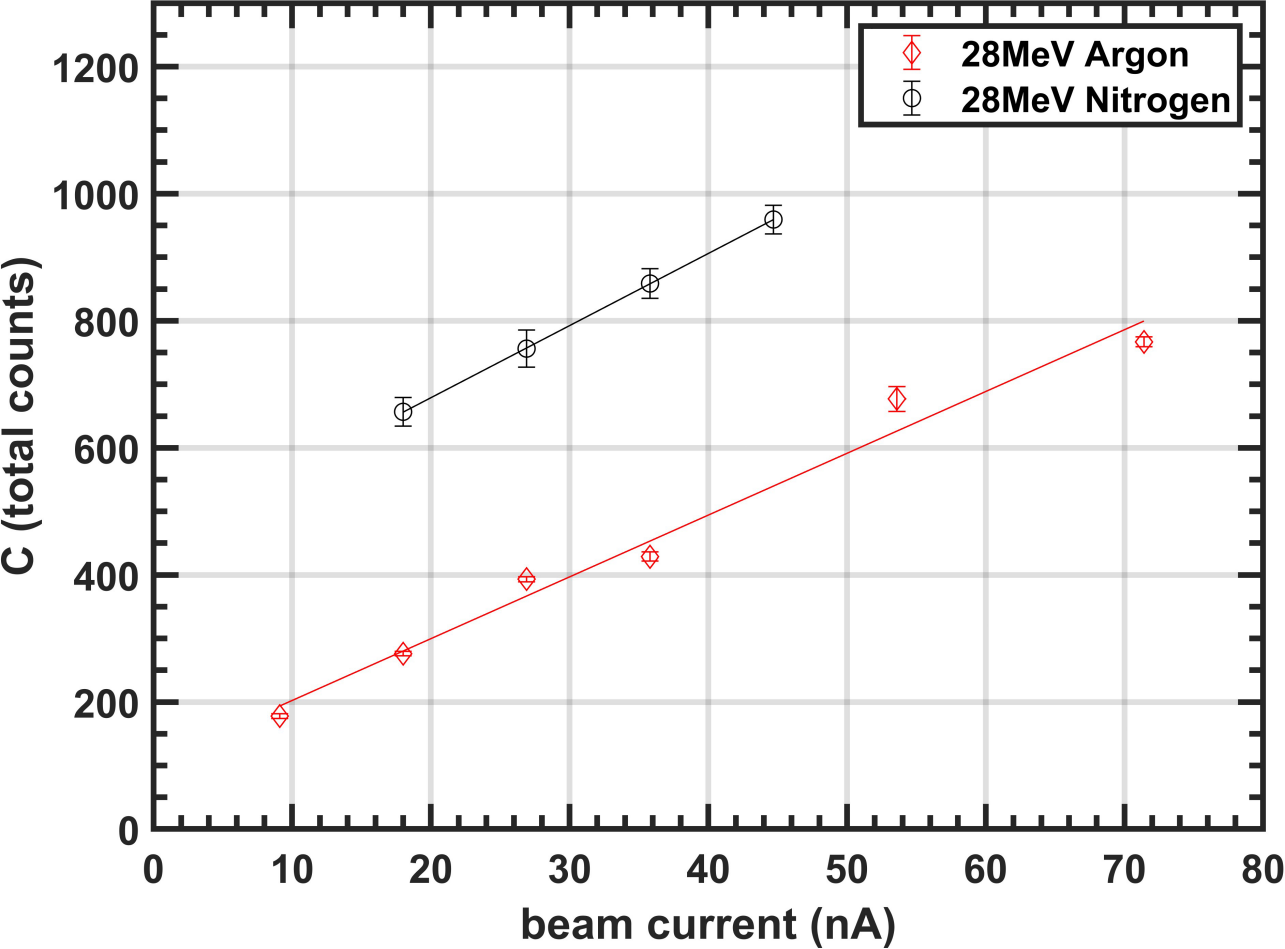
Variation of total counts *C* with beam current for 28 MeV proton beam.

**Figure 5** illustrates the variation in total integrated counts *C* with beam current for a 28 MeV proton beam, measured using both argon and nitrogen gas curtains in experiments conducted at the University of Birmingham. The data follows a predominantly linear trend, consistent with the behavior predicted by Equation 6.

**Figure 6:**
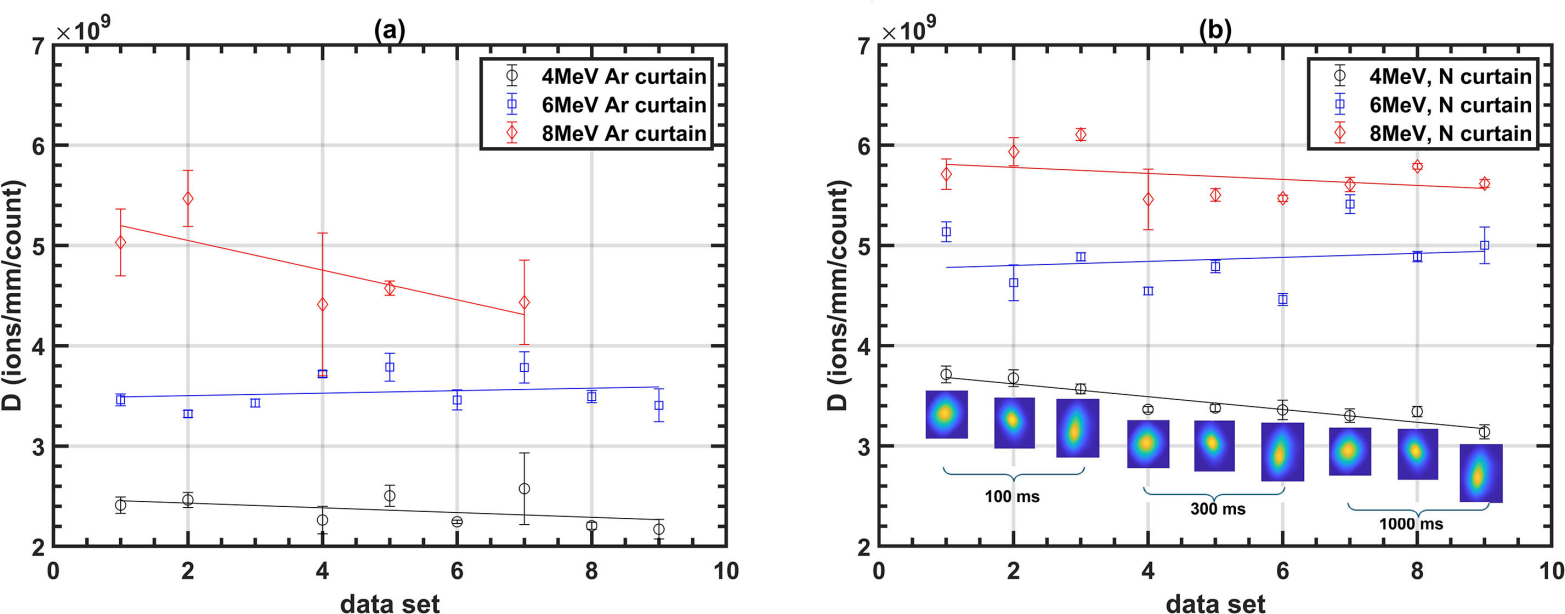
*D* values for different energies at a 100 nA beam current for **(a)** argon and **(b)** nitrogen curtain.

**Figure 6** presents the values of parameter *D* for various datasets at three different beam energies, measured at a 100 nA beam current. The results are shown for both argon and nitrogen curtains. Each data point corresponds to a single dataset, with the markers representing the average value of *D* calculated from 100 images, while the error bars indicate the standard deviation. Each of these datasets represent a different integration time and beam shape. The plots reveal that *D* increases as the ionization cross-section decreases with energy, aligning with the predictions from Equations 3 and 8.

The difference in *D* values between argon and nitrogen curtains arises from differences in their ionization probabilities. Although their ionization cross-sections are comparable at the studied energies, the primary factor influencing ionization probability is the difference in curtain densities which was approximately 2.4 × 10^15^ molecules/m^3^ for nitrogen and 5 × 10^15^ molecules/m^3^ for argon, measured using the method stated in our previous work (15, 18).

Parameter *D* shares similarities with *β*, as both quantify detector sensitivity, but they do so in distinct contexts. While *β* represents the overall sensitivity in detecting the beam, *D* specifically measures the sensitivity at the pixel level. This distinction is crucial because it decouples sensitivity from camera resolution. For instance, doubling the sensor resolution decreases the total count per unit sensor area by a factor of four, but the sensor area decreases by the same factor, effectively cancelling it out. As a result, *D* will remain the same for a different pixel size. Additionally, variations in beam shape affect the particle fluence, resulting in higher counts on a few pixels for a tightly focused beam compared to broader beams. However, the total count *C* remains unchanged, ensuring that both *β* and *D* are independent of beam shape. This is evident in the beam profile insets of **Figure 6b**, which show that the value of *D* remains nearly stable across different beam shapes. The steady fall in the value with data sets is primarily due to random fluctuations in the beam current from its calibrated value during measurements. Notably, the larger errors observed for the 8 MeV beams are attributed to the reduced performance of the image analysis process, which struggles with the weaker signals at this energy.

To compare the gain in detector sensitivity between experiments conducted at the DCF and the UoB, value of *D* for DCF experiments are extrapolated for 28 MeV assuming *D* varies linearly with energy, to match the conditions of the UoB experiments. The extrapolated values compared for both argon and nitrogen jets are shown in **Table 2**. This shows that sensitivity improvements by a factor of 80 was achieved between two sets of experiments.

**Table 2:** Comparison of *D* values between the experiments at DCF and UoB, highlighting the improvements in detector sensitivity.

*D* ^28^ *^MeV^* (fluence/count)	Argon	Nitrogen
DCF experiments (extrapolated)	1.5 × 10^10^	1.75 × 10^10^
UoB experiments	3.8 × 10^8^	2 × 10^8^
Gain (noise normalized)	~ 40	~ 87.5

## 4 Discussion

The previous section quantified the performance of the gas curtain-based beam profile monitor (SGC-IPM) in terms of the parameters *C* and *D*, which vary with beam current and energy but are independent of beam shape. The parameter *D* also defines the threshold fluence required for detection. Since beam fluence can be associated to the cumulative dose instead of the dose rate, it provides a basis for estimating detector performance under various beam parameters, including those relevant to the FLASH modality.

The commonly accepted dose rate for FLASH is >40 Gy/s. The beam parameters that qualify as FLASH are likely an interdependent combination of dose, dose rate, repetition rate, and the number of pulses (19). Therefore, it is challenging to generalize typical beam parameters for FLASH therapy. A useful rule of thumb suggests that delivering 1 Gy of dose to a 1-liter clinical volume, within a depth of 15–20 cm in water, in 1 minute requires a beam current of approximately 0.25 nA (20). This corresponds to delivering roughly 9.375 × 10^10^ protons over the course of 1 minute. To deliver the same dose at a FLASH dose rate of 40 Gy/s, the same number of protons would need to be delivered 2,400 times faster, corresponding to a beam current of approximately 600 nA delivered within 25 ms.

For a case study, we consider a treatment plan designed to deliver a cumulative dose of 4 Gy to a 1-liter volume using spot scanning with a 2 mm spot size within 100 ms (40 Gy/s), similar to a previous study (4). If the SGC-IPM is used to monitor the beam, it is desirable to reproduce the dose distribution for a single layer, or at least, within the entire treatment/target area. Achieving this requires detecting individual spots, ideally within a single energy layer, or all layers combined at the least. The detection threshold of the SGC-IPM is expressed in terms of beam fluence as shown in **Table 2**. The average beam fluence for the above plan for 1-liter and 125ml volume is shown in **Table 3**.

**Table 3:** Beam fluence for different irradiation plans to deliver a dose of 4 Gy using spot scanning.

Dimensions (cm^3^)	Total P+	Layers	Spots	Spacing	P+/spot	Fluence (single layer)	P+/spot (all layers)	Fluence (all layers)
10 × 10 × 10	3.80 × 10^11^	34	96,222	2	3.95 × 10^6^	1.26 × 10^6^	1.34 × 10^8^	4.28 × 10^7^
5 × 5 × 5	5.50 × 10^10^	17	10,622	2	5.18 × 10^6^	1.65 × 10^6^	8.80 × 10^7^	2.80 × 10^7^

The beam fluence for a single layer is two orders of magnitude below the detection threshold of SGC-IPM. However, the beam fluence for all layers combined is below the detection threshold by a factor of 5. If the treatment modality were to deliver 40 Gy instead of 4 Gy, the proton fluence for all layers combined would comfortably fall within the threshold fluence limit, suggesting that, SGC-IPM can record the scanning pattern before a 40 Gy dose is delivered to the treatment volume. While this may not allow precise monitoring of individual spot positions, it would enable reconstruction of the overall spot-scanning pattern. Although 40 Gy is an unusually high dose from a clinical perspective, this example is intended purely to illustrate the detection threshold. This analysis suggests that improving the detector sensitivity by a factor of 40 would reduce the detection threshold to approximately 1 Gy, a more clinically relevant value.

The case study assumes the SGC-IPM is installed at the nozzle, which is technically infeasible due to its size. A more realistic installation location would be before the gantry or along the beamline closer to the accelerator, where the beam fluence is higher to compensate for the transport losses. At these locations, the current sensitivity of the SGC-IPM might be sufficient to reconstruct the scanning pattern. The beam fluence at the installation location would determine how much dose must be delivered before the SGC-IPM can provide information on the scanning pattern, which makes generalization difficult. Nonetheless, this case study provides insights into the performance of the detector in its current configuration and highlights areas for improvement.

Future studies will focus on reducing the threshold fluence requirement by further enhancing the SGC-IPM’s performance. A significant improvement, up to a factor of 10, can be achieved by increasing the density of the gas curtain to 10^17^ particles/m^3^, as demonstrated previously (21). Our current research is also focused on optimization of ion collection using a smaller ionization profile monitor, evaluating beam profile accuracy by comparing results with established methods such as scintillator screens, and generating sufficient experimental data to develop robust scaling factors for accurate calibration and beam current prediction.

## 5 Conclusion

This paper presents a supersonic gas curtain-based ionization profile monitor (SGC-IPM) developed to measure the 2D profile of a proton beam without perturbing the beam. The device was demonstrated to measure beam profiles for proton beams with energies ranging from 4 to 28 MeV and currents between 1 and 100 nA. A mathematical model is introduced to quantify the detector response to different beam currents and energies, based on integrated counts and beam fluence. The model was validated with experimental data, showing that total integrated counts increase linearly with beam current, while the scaling parameter *D* increases with the beam energy, consistent with the reduction in ionization cross-section. Validated model is then used to assess the SGC-IPM’s performance in terms of the sensitivity.

The experiments show that the SGC-IPM exhibits a linear response to beam currents between 1 and 100 nA at 28 MeV, independent of the beam’s shape or size. The sensitivity of the device is defined in terms of proton fluence, to make the response independent of dose rate. Using this, the response of the device is evaluated in the context of a FLASH spot-scanning treatment plan by determining the minimum dose required to capture the scanning pattern. This evaluation highlights the need to lower the detection threshold of the SGC-IPM further to accommodate beam parameters relevant for FLASH-PBT.

## Data Availability

The raw date supporting the conclusions of this article will be made available by the authors, upon request without undue reservation.
